# Pulmonary AL Amyloidosis as a Cause of Interstitial Lung Disease Diagnosed With Transbronchial Lung Biopsy

**DOI:** 10.1002/rcr2.70769

**Published:** 2026-09-22

**Authors:** Kai Wen Hwang, Ju Ee Seet, Michelle Poon, Kay Choong See

**Affiliations:** ^1^ Department of Medicine, Division of Respiratory and Critical Care Medicine National University Hospital Singapore; ^2^ Department of Pathology National University Hospital Singapore; ^3^ Department of Haematology‐Oncology National University Cancer Institute Singapore

**Keywords:** interstitial lung disease, pulmonary amyloidosis, transbronchial lung biopsy

## Abstract

Pulmonary amyloidosis is a rare manifestation of amyloid deposition which can present as a rare albeit treatable cause of interstitial lung disease. Clinical features and radiological findings are nonspecific while bronchoalveolar lavage fluid analysis alone may be misleading, hence tissue diagnosis is necessary to establish diagnosis.

## Graphical abstract

Importance of tissue diagnosis in pulmonary amyloidosis, a rare albeit treatable cause of interstitial lung disease.

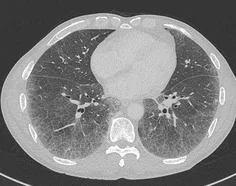

## 1 Introduction

Pulmonary amyloidosis is a rare manifestation of amyloid fibril deposition requiring tissue biopsy for diagnosis [1] as it presents with nonspecific symptoms and radiology [2].

This case highlights the central role of bronchoscopic transbronchial lung biopsy in achieving histopathological diagnosis of pulmonary amyloidosis.

## 2 Case Report

A 61‐year‐old man presented with gradually progressive exertional dyspnoea, unintentional weight loss and bilateral lower limb swelling of 7 months' duration. Coronary angiogram was normal, although transthoracic echocardiogram reported preserved left ventricular ejection fraction with a hint of apical sparing −16% noted on global longitudinal strain imaging and an apical‐to‐base ratio of 0.83 and N‐terminal pro‐brain natriuretic peptide (NT‐pro‐BNP) serially uptrended from 425 to 1026 ng/L.

**FIGURE 1:**
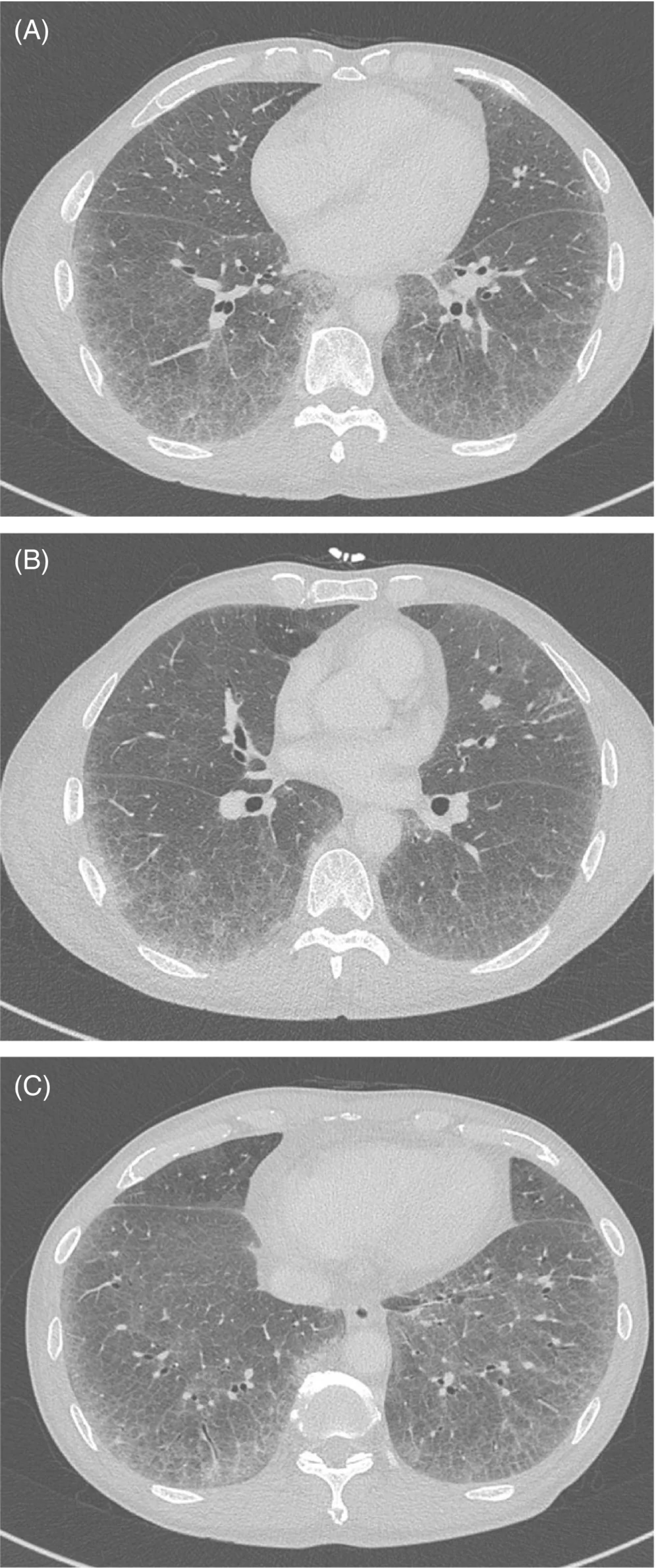
Representative chest computed tomography images. (A) Bilateral lower lobe reticular interstitial markings with interlobular septal thickening, associated ground‐glass opacities and mosaic attenuation in a subpleural distribution, consistent with non‐usual interstitial pneumonia pattern of interstitial lung disease. (B) Scattered nodules with tree‐in‐bud nodularities seen in the lingula. (C) Diffuse cylindrical bronchiectasis with airway wall thickening.

Chest radiograph identified diffuse bilateral mid to lower zone reticular opacities, and chest computed tomography (CT) demonstrated bilateral lower lobe reticular interstitial markings with interlobular septal thickening, associated ground‐glass opacities and mosaic attenuation in a subpleural distribution (Figure 1A) representing a non‐usual interstitial pneumonia (non‐UIP) pattern of interstitial lung disease. Scattered nodules (Figure 1B) and diffuse cylindrical bronchiectasis with airway wall thickening (Figure 1C) were also seen. Serological testing was negative for anti‐nuclear antibody, rheumatoid factor, anti‐cyclic citrullinated peptide, hypocomplementaemia, anti‐double stranded deoxyribonucleic acid (anti‐ds‐DNA), anti‐extractable nuclear antigens, anti‐neutrophil cytoplasmic antibodies and myositis panel antibodies. Pulmonary function testing demonstrated restriction with forced expiratory volume in the first second (FEV1) of 2.01 L (68% predicted), forced vital capacity (FVC) of 2.36 L (64% predicted), FEV1/FVC ratio of 0.85, total lung capacity (TLC) of 4.8 L (74% predicted) and severe gas exchange defect with diffusing capacity for carbon monoxide (DLCO) of 7.48 mL/(min mmHg) (30% predicted). The patient was able to walk 450 m on a 6‐min walk test with no hypoxaemia.

**FIGURE 2:**
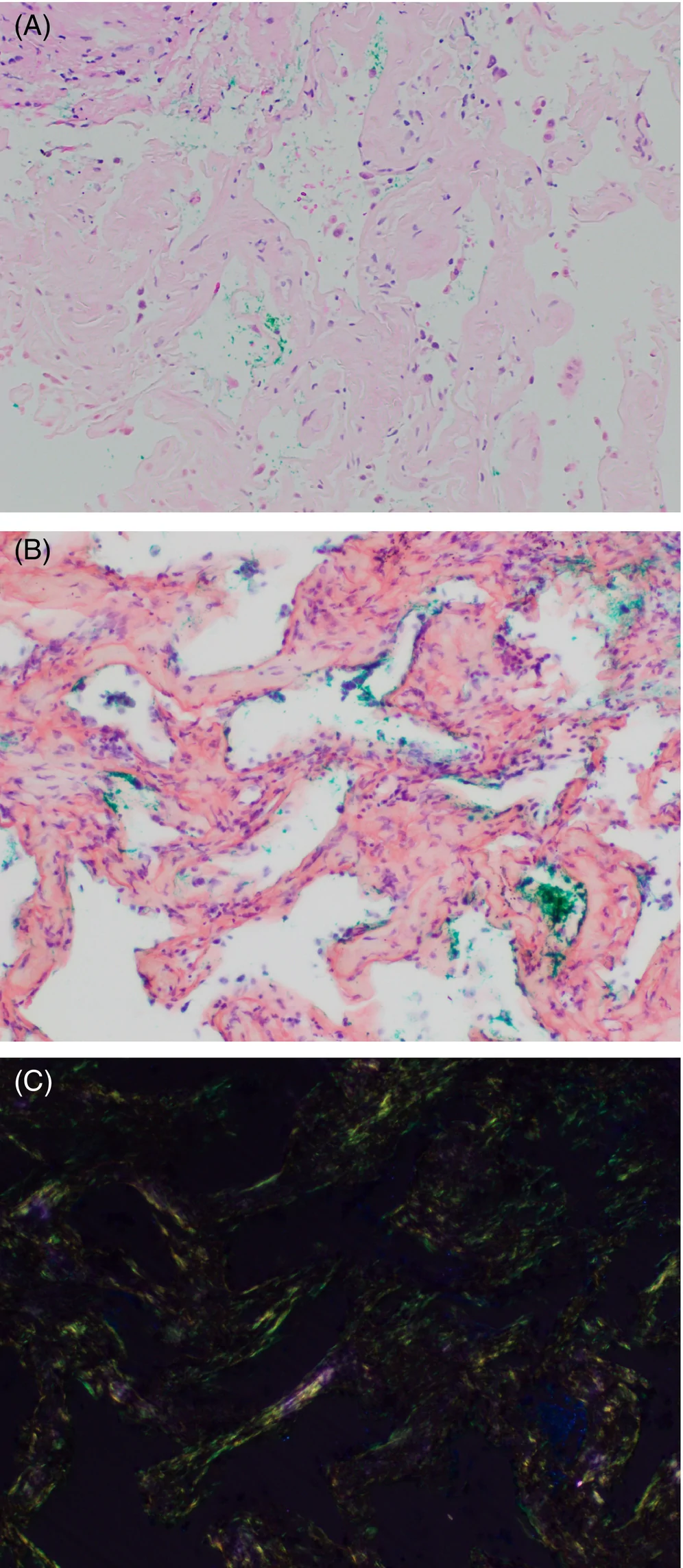
(A–C) Histology of lung biopsy showing diffuse interstitial deposition of pale staining amyloid in the interstitium without significant associated inflammation (A: Haematoxylin and eosin, B: Congo Red (light microscopy) showing salmon‐pink staining, C: Congo Red (polarized light) showing apple green birefringence, original magnification of all slides 100×).

Bronchoalveolar lavage fluid (BALF) of the right lower lobe anterior basal segment demonstrated 146 nucleated cells per microlitre, with 36% neutrophils, 34% monocytes, 26% eosinophils and 4% lymphocytes, albeit potentially inaccurate despite manual counting as the fluid specimen was reported as having cell clumps or debris. BALF cytopathology found normal cellular constituents with no eosinophilia, and microbiological studies were negative for clinically significant pathogens. Histopathology from transbronchial lung biopsy (TBLB) of the right lower lobe lateral basal segment identified diffuse interstitial deposition of amyloid within both blood vessels and alveolar septa (Figure 2A–C). A diagnosis of alveolar‐septal pulmonary amyloidosis was established.

Serum total protein was 53 g/L with albumin 30 g/L. Serum lambda free light chain (FLC) was markedly elevated at 1899.8 mg/L, while kappa FLC was normal at 13 mg/L, producing a lambda: kappa FLC ratio of 0.01. Serum immunofixation electrophoresis detected a band immunotyped as lambda FLC. Bone marrow aspiration and trephine biopsy yielded plasma cell dyscrasia with lambda immunoglobulin light chain restriction, and liquid chromatography tandem mass spectrometry performed on peptides extracted from the biopsy specimen detected AL‐(lambda)‐type amyloid deposition. While bone marrow flow cytometry was suggestive of multiple myeloma, he had no features of anaemia, hypercalcaemia, renal dysfunction, or bone lesions on skeletal survey CT. Cardiac magnetic resonance imaging (MRI) was characteristic of cardiac amyloidosis, including diffuse subendocardial late gadolinium enhancement in the left ventricle basal to mid‐cavity with altered gadolinium kinetics and elevated gadolinium extracellular volume of 46%.

The patient was started on daratumumab, cyclophosphamide, bortezomib and dexamethasone combination therapy over six cycles, before transiting to maintenance daratumumab as treatment of pulmonary and cardiac AL amyloidosis. After completion of intensive‐phase therapy, his symptoms of exertional dyspnoea and lower limb swelling improved with NT‐pro‐BNP downtrending to 248 ng/L. Serum albumin improved to 40 g/L and lambda FLC reduced to a nadir of 2.5 mg/L. His pulmonary function tests did not demonstrate further significant deterioration, with TLC 4.92 L (76% predicted) and DLCO 6.08 mL/(min mmHg) (24% predicted). Serial chest radiographs demonstrated stable bilateral lower zone reticular opacities, with no new abnormalities seen. Given his marked clinical and haematological response to treatment, cross‐sectional thoracic imaging and echocardiography were not repeated.

## 3 Discussion

Amyloidosis involving the lung can present with various nonspecific radiological patterns [2] including interstitial lung disease and requires tissue biopsy to establish diagnosis [1]. In this case, TBLB was instrumental in obtaining adequate tissue for histopathological examination and facilitating timely initiation of quadruple therapy with favourable disease response, greatly aided by multidisciplinary discussion between the pulmonologist, pathologist and haematologist. Relying on BALF interpretation alone might otherwise have led to a misdiagnosis of eosinophilic pulmonary disease [3] given the patient's eosinophil count greater than 25% and bland cytopathology in conjunction with his radiological non‐UIP pattern, which would have delayed appropriate treatment and may have resulted in further disease progression. Notably, overall nucleated cell count was not particularly high; peripheral eosinophils in this case were not elevated at 3%, reported cell clumps might indicate a significant proportion of epithelial cells, and no eosinophils were seen on lung histopathology, highlighting the importance of caution in diagnosing pulmonary eosinophilia on BALF in the absence of peripheral eosinophilia.

While pulmonary amyloidosis remains a rare cause of interstitial lung disease, it is often amenable to treatment when diagnosed, underscoring the importance of histopathological sampling in undifferentiated non‐UIP pattern interstitial lung disease.

## Author Contributions

**Kai Wen Hwang:** writing – original draft, visualisation, data curation, writing – review and editing. **Ju Ee Seet:** resources, visualisation, data curation, writing – review and editing. **Michelle Poon:** resources, data curation, writing – review and editing. **Kay Choong See:** conceptualisation, writing – review and editing, supervision, project administration.

## Funding

The authors have nothing to report.

## Consent

The authors declare that written informed consent was obtained for the publication of this manuscript and accompanying images and attest that the form used to obtain consent from the patient(s) complies with the Journal requirements as outlined in the author guidelines.

## Conflicts of Interest

Kay Choong See is an Associate Editor of Respirology Case Reports and was not involved in peer review and publication decisions of this article. The other authors declare no conflicts of interest.

## Data Availability

Data sharing not applicable to this article as no datasets were generated or analysed during the current study.
